# “I would prefer to be dead than to live this way”: Lived experiences of stigma and discrimination against khwaja sira in Swat, Pakistan

**DOI:** 10.1017/gmh.2024.53

**Published:** 2024-04-30

**Authors:** Sameena Azhar, Imtyaz Ahmad, Maria Mercedes Guzman Herrera, Nadeem Tariq, Riya Lerner

**Affiliations:** 1 Graduate School of Social Service, Fordham University, New York, NY, USA; 2 Department of Political Science, Hazara University, Mansehra, Pakistan; 3 Department of Pakistan Studies, National University of Modern Languages (NUML), Islamabad, Pakistan

**Keywords:** khwaja sira, stigma, discrimination, sexual and gender minorities, third gender

## Abstract

Due to their identification as third gender people, *khwaja sira* have historically been subjected to experiences of social marginalization. However, the extant literature has not fully explored the lived experiences of stigma and discrimination against khwaja sira in the Swat Valley of Khyber Pakhtunkhwa, Pakistan. To address this gap, we conducted 45 interviews with khwaja sira in Mingora, Swat, Khyber Pakhtunkhwa to better understand their experiences of gender-nonconformity stigma and discrimination in various social contexts, including within their families, in accessing health care, and within education and work contexts. Applying Minority Stress Theory and utilizing thematic content analysis, the present study identified three dimensions of gender-nonconformity stigma: (1) internalized stigma, namely feelings of shame and embarrassment; (2) perceived stigma, namely opinions others had of khwaja sira regarding lack of employability or engagement in sex work; and (3) enacted stigma, namely exclusion from families, in educational settings, in religious spaces, and in healthcare settings. Findings should inform future social intervention and community practice engagements with khwaja sira communities in Pakistan.

## Graphical abstract

**Figure:**
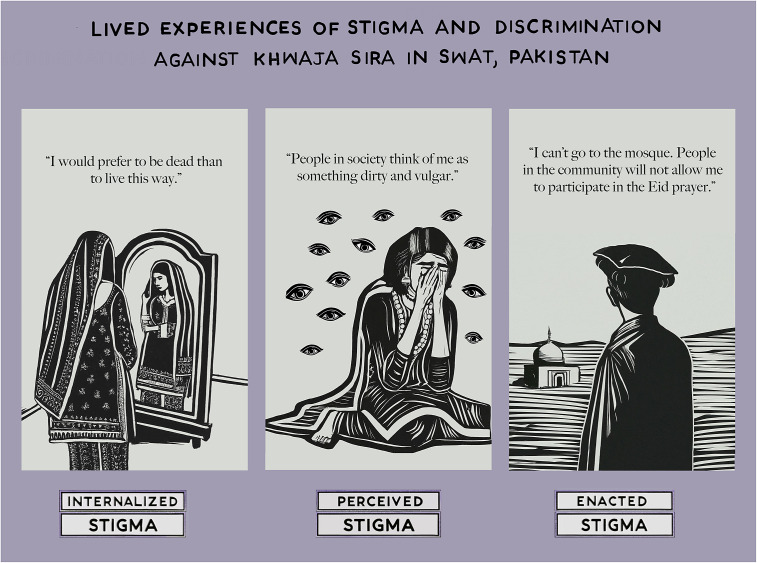
In the present study, we identified three dimensions of gender-nonconformity stigma impacting khwaja sira in Swat, Pakistan: (1) internalized stigma, namely feelings of shame and embarrassment; (2) perceived stigma, namely opinions others had of khwaja sira regarding lack of employability or engagement in sex work; and (3) enacted stigma, namely exclusion from families, in educational settings, in religious spaces, and in healthcare settings.

## Impact statement

Given recent social policy developments in Pakistan, the findings from this study can help inform future social intervention and community practice engagements with khwaja sira (third gender) communities. In 2011, the Pakistani Supreme Court passed the National Data Base Registration Act, permitting khwaja sira and other gender-nonbinary people from legally registering as a third gender on their National Identity Cards, voting registration cards, and passports. In 2018, the Transgender Persons (Protection of Rights) Act of 2018 explicitly listed inheritance, education, and the right to vote as rights, prohibited discrimination against gender-nonbinary people, and ensured khwaja sira access to public spaces and private property. However, in 2023, the Federal Shariat Court of Pakistan declared the Transgender Persons Act 2018 inconsistent with Islamic principles, striking down the self-identification of gender identity and the provision of inheritance rights as violating the tenets of the Qur’an. The Khunsa Persons (Protection of Rights) Bill was introduced with the aim of attaining “consistency with the injunctions of Islam” by denying those rights. Given the recent proposed retraction of legal rights for third gender people in Pakistan, our study findings have relevance for educators, healthcare providers, and community-based practitioners who engage with khwaja sira communities.

## Third gender identities in South Asia

*Khwaja sira* is the term most commonly used to identify third gender people in Pakistan (Khan, 2016), a community who are estimated to number approximately 1.5 million people (Pamment, 2010). Like the term *hijra* (Nanda, 1990; Reddy, 2006), *khwaja sira* is an umbrella term used in South Asia to identify people who have been assigned male sex at birth, but currently identify as neither male nor female, but alternatively as a third gender. Other labels for third gender people in South Asia include *khusra*, *jhanka*, *zenana, murat,* and *kinnar.* The term *murat* combines the words *mard* (meaning man in Urdu) with the word *aurat* (meaning woman in Urdu), but carries a pejorative connotation.

In precolonial South Asia, khwaja sira were highly respected, particularly in the Mughal era, where they held significant responsibilities in the royal courts and harems (Alamgir, 2022; Sher et al, 2022), including roles as advisors and performers. For example, in the early 14th century, Sultan Alaud-din Khilgi appointed a khwaja sira, Malik Kafur, as the Grand Wazir, the effective head of government, and later as the Commander-in-Chief of his army (Junejo, 1994; Gugler, 2011). Similarly, Mahboob Ali Khan, another khwaja sira, occupied an important position during the days of the last Mughal Emperor, Bahadur Shah Zafar as the Mukhtar-i-Kul or Controller General of Revenues (Junejo, 1994).

However, following British colonialism, khwaja sira faced scrutiny for their public song and dance performances, which were perceived as disruptions to public space and decency. The 1871 Criminal Tribes Act and the 1876 Dramatic Performance Act made the daily activities of khwaja sira illegal, namely the acts of singing, dancing, and “cross-dressing”. Khwaja sira were also wrongly accused of crimes like kidnapping and castrating children (Hinchy, 2017). British officials often incorrectly referred to them as “eunuchs,” although many khwaja sira and hijra had never been castrated and sometimes had children of their own. Following Indian and Pakistani independence, the Criminal Tribes Act was repealed in 1952 (Reddy, 2005). However, Section 377 of the 1860 Indian Penal Code continues to criminalize acts seen to be “against the order of nature,” which has largely been interpreted to include sodomy and homosexuality (Bhaskaran, 2002; Hinchy, 2013). While India’s Supreme Court struck down Section 377 in 2018, it still remains part of Pakistan’s Penal Code (Azhar et al., 2023).

Following British colonization, many khwaja sira had limited educational opportunities, which have also influenced their work and financial options (Khan, S., 2017). One means by which khwaja sira earn a livelihood is through *badhai*, or the receipt of donations in exchange for the conferring of blessings at weddings, childbirths, and other special occasions (Khan, 2016; Pamment, 2010). Khwaja sira are seen to be auspicious for the couple or child’s future happiness and fertility, due to their divine associations as androgynous, spiritual beings who hold the ability to bless and curse others (Hamzić, 2019). In addition to badhai, khwaja sira engage in dance performances, sex work, and begging to earn a livelihood (Hambly, 1974).

## Stigma and discrimination of khwaja sira in Pakistan

Khwaja sira have faced social exclusion and oppression in multiple social spheres, namely in school settings, within their families, and in healthcare settings. Within school settings, khwaja sira have often experienced a lack of acceptance due to their nonconformity to traditional masculine gender roles (Alizai et al., 2017; Khan, 2020). Khwaja sira reported that they were identified as feminine boys; as youth, they often played with dolls, used nail polish, and wore makeup (Alizai et al., 2017). Many stated that their teachers would taunt them about their traditionally effeminate behavior and classmates would avoid playing with them (Somasundaram, 2009; Virupaksha and Muralidhar, 2018). They were often bullied in the classroom and ostracized in group social interactions (Somasundaram, 2009; Virupaksha and Muralidhar, 2018).

In terms of their experiences with their families, many khwaja sira reported that they were forced to drop out of school at a relatively young age, due to high levels of social rejection within their families of origin (Mithani and Burfat, 2003; de Lind van Wijngaarden et al., 2013; Khan, 2020). They would run away from or be ousted from their homes, finding refuge in communal homes with other khwaja sira, known as *deras*, with a *guru* [leader] that housed and protected a group of *chelas* [disciples]. Gurus and chelas engage with each other through a system of apprenticeship where the guru serves multiple roles as the khwaja sira’s house mother, landlord, pimp, and advisor (Azhar et al., 2022, 2023; Khan, 2020).

In terms of their experiences in religious spaces, strict religious attitudes regarding heterosexuality and traditional gender roles may contribute to negative attitudes toward khwaja sira (Jami and Kamal, 2015). Khwaja sira may suffer mental and psychological problems as a consequence of being socially rejected by mainstream society (Shahzadi and Ali, 2017). Negative attitudes toward queer communities may arise from religious beliefs that view the community’s engagement in gender-nonconformity or sex work as *haraam* [a sin in Islam] (Jami and Kamal, 2015). Similar to our own findings, in previous research conducted in Lahore, Karachi, and Islamabad, khwaja sira reported that as youth, they had been shamed by community members for their gender identities and/or for engaging in sex work (Hamzić, 2019).

Regarding their experiences with healthcare systems, khwaja sira in Pakistan have reported lack of support, minimum income to afford private health care, and distrust of healthcare providers (Ahmad et al., 2024). Khwaja sira have reported experiencing discrimination at low-cost, private clinics by being less likely to be provided medication, more likely to be offered an unnecessary injection, more likely to be dispensed cough medicines, and more likely to be prescribed antibiotics (Rashid et al, 2022). Many have turned to traditional healers due to past negative experiences and financial constraints (Zafar and Ahmad, 2022). Older khwaja sira struggle with health challenges due to social discrimination and marginalization (Rashid et al, 2022). These challenges include psychological stress stemming from stigma and discrimination, social isolation, lack of social support (Arshad et al, 2022), lack of access to equitable medical treatment (Azhar, Dean et al., 2024), and lack of familial support.

## Theoretical framework

Stigma refers to society’s treatment of individuals or groups on the basis of what is considered to be different or deviant, resulting in a marginalized identity and devalued status for the stigmatized individual (Goffman, 1963). The process of stigmatization can involve overt experiences of discrimination, processes of being labeled as “other,” and the attribution of unfavorable qualities, such as being viewed as immoral or promiscuous (Link and Phelan, 2001). Characterized by exclusion, rejection, and blame, stigma often results in experiences of social judgment against the targeted individual or group (Scrambler, 2009). Gender-nonconformity stigma describes the experiences of individuals whose gender identity, gender role, or gender expression differs from the sex they were assigned at birth (Coleman et al., 2012).

The theoretical framework that guides the present study is Minority Stress Theory (MST). One of the central tenets of MST is that sexual and gender minorities are at increased risk for psychosocial stress due to their stigmatization in a heterosexist society (Meyer, 2003). The conceptual model that guides the present study focuses on these three dimensions of gender-nonconformity stigma that are highlighted in MST: (1) internalized stigma, (2) perceived stigma, and (3) enacted stigma (Masa et al., 2022; Meyer, 2003; Zelaya et al., 2008).

Internalized stigma refers to the individuals’ incorporation of society’s negative views regarding gender-nonconformity into their own identity (Ramirez-Valles et al., 2013), particularly focusing on instances of embarrassment or shame (Herek, 2007). This shame can be associated with feelings of worthlessness, low self-esteem, depression, and social isolation. Perceived stigma refers to concerns regarding how a particular condition is viewed more generally by society (Palomar et al., 2013), or rather how people perceive a condition or identity that is deemed to be socially taboo. Social attitudes regarding gender-nonconformity stigma can have significant and deleterious psychosocial impacts (Kota et al., 2020) on the life trajectories of third gender people. Enacted or experienced stigma refers to the experience of prejudice, bias or discrimination as a consequence of being associated with an undesirable group or behavior (Parker and Aggelton, 2003), such as being a third gender person. Analyzing experiences of gender-nonconformity stigma through these constructs can help to better understand how khwaja sira are treated in various social contexts.

### Study objective

Several studies have documented the experiences of stigma and discrimination of transgender and gender-nonbinary individuals in the United States and Western Europe (Nemoto et al., 2011; Reisner et al., 2015). Additionally, an evidence base is emerging regarding stigma and discrimination against khwaja sira in the Pakistani context (Alizai et al., 2017; de Lind van Wijngaarden et al., 2013; Hamzić, 2014, 2019; Miller and Grollman, 2015). However, little research has addressed khwaja sira communities in the northwestern Swat Valley. Swat is situated in the Malakand region of Khyber Pakhtunkhwa (KP), Pakistan and shares a border with the eastern province of Nangarhar, Afghanistan. The Federally Administered Tribal Areas, where Swat is situated, represent one of the most politically volatile regions in Pakistan.

Due to a history of strict gender roles (Kessler and McKenna, 1978) and several decades of social and political instability with neighboring Afghanistan, the Swat Valley provides a unique sociocultural context for the study of gender-nonconformity stigma. Additionally, other studies have not examined stigma and discrimination of khwaja sira in Pakistan across multiple settings, namely at work, within their families, and within institutions. For the present study, we aimed to qualitatively analyze the experiences of stigma and discrimination against khwaja sira in the city of Mingora, located in Swat, Pakistan, within multiple contexts, namely within their families, in accessing health care, and within education and work contexts. While there are multiple stories of resistance and resilience, these themes will be presented in a forthcoming manuscript. This article specifically focuses on experiences of stigma and discrimination against the khwaja sira community.

## Methods

### Study design

This study was a qualitative investigation into the lived experiences of stigma and discrimination experienced by khwaja sira. We recruited 45 khwaja sira in Mingora, Swat. Pakistan to participate in in-depth qualitative interviews. Inclusion criteria for study participants were: (1) self-identify as khwaja sira; (2) resident of Mingora, Swat, Khyber Pakhtunkhwa, Pakistan; (3) proficient in speaking Pashto; and (4) over the age of 18 years.

### Recruitment

Before beginning fieldwork, the research team solicited the approval of local *gurus* (senior leaders) in order to have their *chelas* (disciples) participate in the research study. Following a community-driven discussion, the research team decided that each respondent would be provided a financial incentive of 500 PKR (approximately $2.50 USD at the time of study completion). Research indicates that even small monetary value incentives create statistically significant increases in the response rate for research participation (Abdelazeem et al., 2022). To safeguard against potential coercion of research participation, we ensured that the amount that participants received was less than what they would have earned through other forms of labor, namely sex work. Convenience and snowball sampling were used to recruit study participants from each of the six major *deras* in Mingora. A total of 15 individuals (25% of the total recruited population) were approached but refused to participate, mainly due to concerns regarding confidentiality or incentive for research participation. The final sample size of 45 individuals was determined once the saturation of themes had been reached.

### Data collection

In order to ensure confidentiality, the setting for the interviews were in the office spaces of partnering NGOs or in other private settings in Mingora, Swat, Pakistan. Interviews were conducted by a graduate research assistant, based in Mingora between 2019 and 2021, who had existing research collaborations with the local khwaja sira community. The research assistant was trained in interviewing techniques and research ethics guidelines. All individuals who were involved in the study completed a CITI certification for research ethics. For the interview, open-ended questions were utilized to elicit descriptions of lived experiences of khwaja sira. The interview guide was organized around five domains: (1) *guru-chela* culture, (2) social exclusion, (3) gender-nonconformity stigma, (4) financial relationships, and (5) psychosocial stress. The themes addressed in this article primarily pertain to the second and third larger themes on social exclusion and gender nonconformity stigma.

### Data analysis

All interviews were digitally audio recorded, then subsequently translated and transcribed directly from Pashto into English. Transcripts of the interviews were analyzed using thematic content analysis (Krippendorf and Bock, 2009); inductive analyses were used to identify new themes that emerged from the data. After reviewing the first 10 transcripts, five evaluators – the principal investigators, two research assistants in Pakistan, and two U.S.-based research assistants – developed a codebook of themes. Relevant sections from the transcribed data were assigned codes (Miles and Huberman, 1994). Identified codes were placed in broad groupings, and transcripts were analyzed for common themes. The research team engaged in a weekly, iterative process to group, redefine, and analyze themes and subthemes emerging from the data analysis. Consensus was reached on all coding decisions.

## Results

Applying MST, we identified three main themes regarding the experiences of stigma and discrimination experienced by khwaja sira in Swat, Pakistan: internalized stigma, perceived stigma, and enacted stigma. These themes are visually depicted in our graphical abstract. Under the theme of internalized stigma, we identified the following subtheme: feelings of shame and embarrassment. Under the theme of perceived stigma, we identified the following three subthemes: (1) perceived stigma regarding lack of employability, (2) perceived stigma regarding engagement in sex work, and (3) perceived stigma against one’s family for being associated with khwaja sira. Under the theme of enacted stigma, we identified the following four subthemes: (1) exclusion from families, (2) exclusion in school settings, (3) exclusion from religious spaces, and (4) exclusion from health care. While other literature has examined experiences of stigma and discrimination among sexual and gender minorities in Pakistan, a novel finding from our study is the recognition of intersectional forms of stigma that impact the khwaja sira community. Sources of this intersectional stigma include gender nonconformity stigma, sex worker stigma, and HIV stigma. We will explore the internalized, perceived, and enacted stigma of khwaja sira, highlighting the ways in which these forms of stigmatization intersect to create webs of oppression.

### Internalized stigma

#### Feelings of shame and embarrassment

Multiple respondents shared that they felt a strong sense of internal shame and embarrassment regarding their gender identity and for engaging in sex work. However, because of limited alternative employment opportunities, they felt that they had no option but to continue to engage in sex work.

*I want to limit my earnings and quit sex work and other dirty activities in my life. But this bloody culture has forced me to continue my life by doing this.*

*I wish I could leave this damned, dirty place and start living a respectable life!*

*I can’t get my freedom from this shameful identity of being a khwaja sira.*

These narratives also reveal how culture itself is a powerful drive in limiting livelihood options, propelling khwaja sira to be restricted to taboo avenues of employment, namely begging and sex work.

#### Selective disclosure of gender identity

Many khwaja sira used terms like “dirty” and “shameful” to describe their engagement in sex work. The use of this language echoes themes of stigmatization that are prevalent in Pakhtun society, reinforcing how study participants experienced feelings of decreased self-esteem and self-worth as a result of their gender identity and occupation. These internalized feelings of shame often kept participants from disclosing their gender identity to others in their family or within their social circles.

*I hide my khwaja sira identity at home and in society, where I identify as a male. I haven’t disclosed my third gender identity publicly. In the public eye, I act as a serious man. When I’m at home, I don’t shape my eyebrows or keep my head covered. I tell my family that I work in a hotel in Peshawar.*

Study participants recounted their experiences of concealing their identities or their engagement in sex work in order to protect themselves from experiencing further discrimination or violence. These acts of secrecy and vigilance may serve to prevent khwaja sira from experiencing further social ostracization, particularly in politically unstable environments like the Swat Valley. While participants may have protected themselves by not disclosing their gender identities to community members, they alternatively had to deal with the burden of increased vigilance associated with gender identity concealment.

*People in society think of me as something dirty and vulgar. No one likes to meet me, shake hands, eat, sit or walk together. Everyone thinks of me as a dirty thing, not a human being. I don’t remember anyone in society who honored me. Everyone tries to hurt and disrespect me. People everywhere look down on me and call me dirty names. How can I tell you how my heart feels? How am I forced to live a life worse than a dog as a khwaja sira in this society?*

For some participants, the stress of everyday life had impacts on their mental health.

*I would prefer to be dead than to live this way. I’d prefer to rest in peace and run away from this violent world.*

Responses like these from participants demonstrate the magnitude of psychological distress that resulted from these repeated acts of stigma and discrimination over time.

### Perceived stigma

#### Perceived stigma regarding lack of employability

Khwaja sira shared their experiences of perceived stigma when searching for work in the formal labor sector. Participants reported that their identity as third gender people was associated with a public perception of their inability to be professionally employed and resulted in discriminatory practices against them.

*In regard to having a job, society does not tolerate us being third gender. We can’t adopt any occupation in society.*

*If we have any respectable profession, they will not work with us. When we operate a business, no one will work with us.*

Participants discussed their struggles with finding employment, despite having skills in cooking, tailoring, cosmetology, or hairstyling. They stated that business owners felt that they were unemployable or that they would dishonor their business. Many discussed how their lives changed after joining the *pan*, or the khwaja sira lifestyle, which offered them greater freedom from restrictive gender norms.

*I am great at doing make-up. Due to the taboo nature of my khwaja sira identity, I can’t make that my profession here in Pakistan. I want to go abroad and work in that profession.*

*I can tailor women’s clothes. After joining the pan, I adopted that as my profession, but I couldn’t continue doing that because of the way that people discriminated against me. I left that job and came back to the hijra pan. Here sex work and dancing are the only skills through which I can earn money for my survival.*

A smaller group of khwaja sira reported that they previously worked in mainstream jobs, but were forced to leave their places of employment because of the sexual harassment they experienced in the workplace. Khwaja sira were often targeted by their coworkers for their effeminate behavior as they were seen to be “a doll for their pleasure.”

*I started working in a tailor shop while as a student. I wanted to learn tailoring skills, but there were people who did not leave me in peace. My younger brother was also working with me in that shop. The boys of that village would come to the shop and they would use vulgar language against me. They would harass and abuse me in front of my younger brother. They treated me like a doll for their pleasure. I couldn’t tolerate all of this, so I left that job too.*

Another participant reported having a degree in electrical engineering, but was unable to maintain a job in that field, due to ongoing sexual harassment and bullying.

*When I went to school and college, people in society made fun of me. They used to call me different names and they made my life difficult. This was really difficult for me to tolerate. I thought that when I completed my education, the situation would change. Perhaps once I completed my diploma in electrical engineering, I could join a company. When that time came, I faced more hatred than I did in my student life.*

Because of these experiences of bullying and sexual harassment in other professions, khwaja sira were more likely to rely on dance performance, sex work, and begging as their primary means of income – all professions that are also associated with social stigma, again creating intersectional forms of stigmatization.

#### Perceived stigma regarding engagement in sex work

Numerous participants reported that their experience of discrimination stemmed from khwaja sira being viewed as “dirty” due to their engagement in sex work. Associating with khwaja sira was seen as intermingling with both literal and figurative filth. Khwaja sira reported that people purposefully avoided them in the community and did not want to get physically near them to avoid this association with dirtiness.

*When I engage in sex work, people treat me like a dog. They think I’m selling my body and that I’m dirty. They don’t know why I’m doing it.*

Some reported that others felt that by associating with them, they would be exposed to immoral habits and deviant tendencies. However, a minority of participants reported that they were content with engaging in dancing and sex work as their profession, as it was intertwined with their identity as khwaja sira.

*Dancing and sex work has become part of our nature. If we adopt other respectable professions, we can’t just leave dancing and sex work. It is part of who we are! If you give me a respectable profession to feed myself and fulfill my basic needs, my ass would still never stop dancing! This has become part of our nature because society does not tolerate us in other spaces.*

This participant’s response is indicative of the resilience among khwaja sira communities, in spite of, or perhaps because of, the immense hardships that have been systematically constructed against them in obtaining employment in a “respectable profession.”

#### Perceived stigma against one’s family for being associated with khwaja sira

Perceived stigma also impacts the families of khwaja sira. In addition to hiding their gender identity from their families, multiple respondents reported that they chose not to go home to avoid the stigmatization that their families would experience from being associated with them.

*My family doesn’t know that I’m a part of the hijra pan. If they knew, they would kill me. People would slander our name and say that we are a dirty family because there is a hijra in our family– me. I will become a symbol of shame for them. My family will be excluded from society if they decide to keep a relationship with me. Their only choices would be to kill me or to keep me locked up in the house.*

In these instances, the families are shamed for their association with khwaja sira and are pressured to enact punishment on their family member for the transgression. This leads khwaja sira to feel ashamed, reinforcing existing family schisms.

*It’s been almost two years that I haven’t been able to see my family and I haven’t returned home. I live like a traveler in my own country. Because I’m a khwaja sira, I have to keep my identity a secret. I have to hide all the time. If people in society find out that I am a khwaja sira, they will make my parents and family members the target of their hatred. No one will marry my sisters. Because of this, I have been forced to live far away from my younger brothers too.*

In order to keep the family reputation safe and to protect their family’s honor, many khwaja sira moved to larger cities and joined a community of other khwaja sira in a dera. Many of these khwaja sira either chose to never visit their families at all or their families prevented them from returning home. Furthermore, the family members of khwaja sira experienced harassment and exclusion from social gatherings as well.

*When I joined the hijra pan, people in society started to scorn my brothers because I am hijra. When we go outside the house, everyone in society tells my brothers that they can’t go to the mosque, the hujra [sitting rooms where male members of the community gather to socialize], the market and other public places and gatherings. They can’t attend any birth ceremonies or funerals because of me. People hate us. They don’t want to meet with us. They question my family about why they haven’t killed me because of the activities in which I participate. They don’t want their children to live near me. They will say, “Your brother is a hijra and he is dancing in front of people at that place and he attends sex parties. People do dirty things with him and you live with your children with him! Shame on you!”*

Some reported concern over their children not being able to find partners as a result of the shame brought upon by having a khwaja sira in the family.

*The family cannot even tolerate me at their home because they think that I am a disgrace for all of them. If anyone saw me with them, they will look at my family with contempt and hatred. My darling young daughters are sitting at home and their beautiful youth is passing by, without them being married. No one, not even a poor man, is willing to marry them, because their only fault is that their father is a khwaja sira.*

One khwaja sira reported that their family members were forced to move to another town because of the negative associations connected to them, fracturing the family’s ties to one another. These instances of having to perpetually worry about what others thought about them were commonplace, and created the conditions for khwaja sira to be further alienated from their families and communities.

### Enacted stigma

#### Exclusion from families

While some khwaja sira reported that they were in good relationships with their families, several other khwaja sira reported that they were forced to hide their gender identity from family members to protect their own sense of self-worth, to prevent themselves from experiencing violence within their families, or to protect their family’s honor. Several participants reported how their family members were unaccepting of their gender identity if it were to become publicly known.

*They don’t know about my job nor about my khwaja sira identity. When I go home, I dress up as a man. I wear men’s clothes, put a hat on my head, and take my male shawl with me. I make myself look like a traditional man… I tell them lies about how I work in a shopping mall. Thanks to almighty Allah we are living a pleasant life, but they don’t know about me joining the pan. Once they know, that will create problems for me and make my life difficult.*

Living a life of secrecy then becomes necessary in order to protect both themselves and their families from further shame or ostracization.

*When I come back home, late at night, I quietly go to my bed. If my mother saw me and asked me again where I had gone, and if it was for that “sexual job”, or why I was coming home at such a late hour of the night, I would ask her to keep quiet. Because if my brothers and my father knew about who I am, they would beat me again.*

In the above instance, a mutual process of denial between this participant and his mother serves to protect a certain sense of family honor. Rather than being seen as acts of secrecy, selective disclosure often serves to protect khwaja sira from experiencing violence or social exclusion within their families.

Participants who had family members who were aware of their gender identity reported that they were often not invited to family events. Family members often engaged in exclusionary behaviors toward them in attempts to correct the individual’s behavior or to set their child “straight.” Acts of violence, including being beaten and being tied up in ropes, were described as punishments for expressing gender-nonconforming behaviors.

*My life with my family is full of pain. They have always been violent towards me. They give me different types of punishments. All the men in the family have beat me. My father, brother, uncles, grandfather, and even my own mother have beat me several times, just so that I would not join the hijra pan. Now all of them hate me. They all consider me to be a source of shame on the family.*

*My family did everything they could to stop me. They tried to kill me because they considered me to be shameful for them forever. My family charged a FIR [First Information Report for a criminal event] on me under the Pakistan Penal Code and sent me to lockup.*

Additionally, multiple khwaja sira reported how hair became an outward symbol of femininity for them and a primary means for their gender expression and performativity. Growing their hair out long became an immediate marker of their third gender identity. For many khwaja sira who concealed their identities from their families, the growing of long hair also became a deterrent to returning home. By returning home with long hair, they would immediately be identified as a khwaja sira. This forced individuals to make difficult decisions regarding whether they should cut their hair in order to be able to visit or reunite with their families, thereby sacrificing a key element of their gender identity.

*My elder family members are very rigid in nature. Last year, I went home and my hair was a little bit long, not like a lady’s hair, but down to my shoulders. My father and brother had just returned from Saudi Arabia. On the second day, they cut my hair and told me to not act like a hijra. They said, “You are a boy and you should behave like a man.”*

*Usually, I go home every six months, but now I’ve grown out my hair, so I can’t go home. Next Ramadan will mark two years since I’ve been home. I intend to go home next Ramadan, after cutting my hair. I have spent a lot of money to take care of my hair. I love my hair the most, out of any part of my body. But what can I do? Respect for parents and family also has value, which is many times more important than my own desires. I am ready to make any sacrifice for their honor.*

Participants repeatedly reported how essential their long hair was to their identity as a khwaja sira. Therefore, choosing to cut one’s hair becomes a symbolic demonstration of giving up their gender identity, their lifestyle, and their comfort – either temporarily or permanently.

#### Exclusion in educational settings

Many participants described being sexually harassed in school, often by teachers and other students. Participants described persistent harassment from fellow students and teachers based on their gender-nonconforming behaviors.

*When I was young, I behaved like a girl. That’s why people identified me as a khwaja sira, and there is so much social stigma attached to the khwaja sira identity. Because of that stigma, I couldn’t continue my education and that was the main reason I dropped out of school. When I went to school and returned home, boys would shout behind me and say I looked like a hijra and where was this ‘murat’ going. They used dirty language towards me. I couldn’t come out of my house. Sometimes they stopped me on my way to school and harassed and abused me.*

On their way to school, children with gender-nonconforming tendencies became the object of derision and ridicule. To cope with this persistent bullying, some youth reported engaging in coping behaviors, like taking different routes to avoid the people teasing them on the way to school. Many reported that they were molested by teachers, classmates, and students.

*When I went to school and college, people already knew that I was a khwaja sira… I faced very difficult situations during that time. I can’t go to school the same way every day. Every day I would go a different route, to protect myself. Different groups of boys and people waited for me on my way. They would have kidnapped me by force and would have sexually abused me… Inside the class, boys used to put their fingers in my ass, harass me, and abuse me. I couldn’t go to the bathroom. They would follow me. I couldn’t sit in class. I couldn’t continue my studies in that situation.*

*I faced a lot of discrimination during my time in school up to the 10th grade. People used my body like a toy for their pleasure. Even a mulvi (religious leader) molested me by touching my butt. Other teachers and students would sometimes finger me and try to kiss me. When I complained to my father, he was ashamed. He shouted at me, arguing that there must be something wrong with me, asking why I walked and talked like a khwaja sira. He said I should just act like everyone else.*

Due to discriminatory treatment and abuse faced in school settings, many participants were ultimately not able to complete a primary level of education.

*I left school because they all treated me badly, both boys and girls. People would follow me and curse at me. They would say that I look like a hijra and I would cry. I dropped out of school and became a khwaja sira.*

Without being able to continue their education, khwaja sira had limited financial or employment opportunities, again propelling them to engage in sex work as a means of livelihood.

#### Exclusion from religious spaces

Khwaja sira often experience exclusion from religious spaces, namely in mosques. For example, while attending prayers at a mosque, some participants reported that others made disparaging remarks about them.

*My fellow khwaja sira face much higher levels of stigma and discrimination than here. They can’t go to the mosque. When they go to the mosque for prayer, people use very bad language and cuss at them, which no one can tolerate. People have told them in front of all the people in the mosque in the village, “Look, that person who fucks around is now coming here to the mosque to pray.” No one in society wants to let hijra stand, sit, talk with them, or allow them into the mosque or any other public place in the village.*

Others reported being barred from entering the mosque at all or from attending the funerals of their own relatives.

*I can’t go to the mosque because I’m a khwaja sira. On the occasion of Eid [a major Muslim holiday], I wanted to go pray when I was home, but people in society will not allow me to offer the Eid prayer at the mosque.*

*I can’t participate in religious events like Eid and so on. And I also can’t participate in weddings and other cultural events… Other villagers will never accept me for being a khwaja sira. When my grandmother died – I still love and miss her very much – I would go at night to see her body and come back at night because I couldn’t participate in her funeral ceremony and prayers.*

The shame of the family being associated with a khwaja sira is so strong that some khwaja sira reported wanting to maintain secrecy regarding their gender identities, even following their own death.

The typical washing of the corpse that takes place prior to an Islamic burial (*ghusl*) is gender-segregated, such that men wash the bodies of other men and women wash the bodies of other women. Khwaja sira are left out of this ritual as there is no appropriate gender group to wash their bodies in the mosque. Additionally, some religious leaders may refuse to perform the death and funeral rights for a khwaja sira who was a *nirban* (had their penis/testicles removed), as these surgeries are sometimes viewed to be *shirk* or sinful acts. Therefore, the act of having this surgery performed and being identified as a nirban also carries a certain level of stigma. The failure of having a decent death inflicts further shame on the family and denies the individual and their family a proper burial.

*Even if I die, I wouldn’t want someone telling my family that I was a khwaja sira.*

*I am concerned about my respect and the social status of my father in society. If I was nirban [had my penis and testicles surgically removed] and I died, I would be afraid of my body being cleaned by my family. I couldn’t imagine the situation my parents would have to face and the shame that would fall upon them.*

In death, khwaja sira who have had gender reassignment surgeries would be physically identifiable as being third gender and would not have an appropriate gender group to wash their bodies for them. They would no longer be considered men (nor women), and would be excluded from the process of a decent death. In fear of such an indecent death, some khwaja sira may refrain from having gender reassignment surgery at all.

#### Exclusion from health care

Several participants reported delaying or avoiding the receipt of health care in order to protect themselves from experiences of discrimination from medical professionals or hospital staff. Hospital staff often lacked awareness of the medical needs of khwaja sira, opening them up for experiences of maltreatment and leaving khwaja sira to feel as though they were being examined and observed, like exotic animals.

*Being a khwaja sira, when I go to the hospital or any other medical care setting, people quickly gather around me. They consider me to be something very unique and unnatural, like a dangerous animal without a tail or a head… This is not only the behavior of the general public, but the hospital staff too. The state should provide separate hospital wards and medical care settings for khwaja sira, like they do for men and women.*

Some participants reported that their healthcare provider would sexually harass them as well. To avoid these experiences, some khwaja sira ensured that they had a witness with them to protect themselves from inappropriate touching or questioning.

*There has never been a doctor in my life who has not looked at me with disgust or treated me the wrong way. Every time I go to the doctor, he begins by making fun of me, then leaves. After being free with me, they put their fingers in my ass. I am so fed up with the attitude of the doctors that now I often catch someone while I’m on my way to a medical appointment and pay them to come with me for my own safety.*

Hospitals in Pakistan are typically gender-segregated into male and female wards. This binary gender divide within social institutions literally provides no room for third gender people. Participants described experiences of being treated with confusion by staff who were not sure if they should be treated in the men’s ward or in the women’s ward, leaving their medical care delayed or altogether avoided.

*Once I was ill and when my fellow khwaja sira brought me to the hospital, we went to the female ward and the hospital staff told us to go to the male ward. When we went there, they told us they couldn’t admit me here because I was not male. A lot of time was spent in figuring out where exactly we had to be. I couldn’t tolerate the pain in my stomach anymore.*

*When we go to the government hospital, there are no wards for us khwaja sira. They admit us with the men, but people inside the hospital still harass and abuse us. They use dirty language when talking about us. A couple of months ago, I went to the government hospital. When the doctors admitted me in male ward, there was a group of people who were crying behind me, saying, “Come and look at this hijra! Someone fucked him so hard, he had to go to the hospital.”*

While the creation of a separate ward solely for khwaja sira may be impractical in terms of numbers of khwaja sira served in localities, hospitals may alternatively move toward the creation of wards that are not segregated by gender at all in order to ensure the inclusion of third gender people in accessing health care.

## Discussion

Globally, gender-nonbinary individuals face significant social disadvantages in education, employment, housing, and health (Nemoto et al., 2011; Reisner et al., 2015). Abuse involving third gender individuals often remain undocumented and unaddressed (Fatima et al., 2022). Khwaja sira in Khyber Pakhtunkhwa, Pakistan have faced social exclusion and limited opportunities throughout their lives, including family and social rejection (Azhar et al. 2023; Azhar, Ahmad and Tariq, 2024).

For the khwaja sira community in Swat, there are additional challenges related to its location in a rural setting, relatively near the Afghanistan border. Ethnic and communal tensions may exacerbate experiences of marginalization and further unequal resource distribution (Khan, 2019), particularly as Afghan refugees have migrated en masse to the Swat region. As of Oct. 3, 2023, the United Nations High Commissioner on Refugees (UNHCR, 2023) reported that 3.25 million people are currently internally displaced by the Afghanistan-Pakistan conflict. The massive influx of Afghan refugees in the Swat valley has created a social, political, economic, and environmental crisis with Afghan refugees being scapegoated as “the main source of anarchy and sectarian differences among the local people” (Mulk et al., 2020, p. 37). Over the past year, there has been a rise in violence in KP, affiliated with the militant Pakistani group, Tehreek-e-Jihad, which is believed to be an offshoot of the Tehreek-e-Taliban Pakistan, or the Pakistani Taliban (Al Jazeera, 2023). Nonprofit organizations and government services may not be as readily available in humanitarian crisis settings, like Swat. Although we are unaware of comparative studies looking at urban versus rural environments, attitudes toward third gender communities may also be less inclusive in rural and militant environments, like Swat, over urban settings, like Karachi or Lahore.

Khwaja sira experienced significant levels of discrimination and violence based on gender (Pamment, 2019), which were exacerbated during the COVID-19 pandemic. In Peshawar, KP, khwaja sira reported experiencing social exclusion, job scarcity, homelessness, and mental health issues (Shah et al., 2023). A recent study revealed persistent discrimination against khwaja sira in Pakistan’s legal system (Alamgir, 2024). Cases involving hate crimes against the khwaja sira community in Peshawar remain unreported or are withdrawn, creating a cycle of impunity and hindering the pursuit of justice (Alamgir, 2024).

In family settings, khwaja sira also experience heightened discrimination, social marginalization, and violence (Alizai et al., 2017; Greenberg, 2012; Ullah et al., 2020). While the family serves as the locus of primary socialization and the forming ground for group identity, it is also often the site for preliminary experiences with gender-nonconformity stigma (Narendran et al., 2021). As evidenced by our findings, the family is often a strong source of enacted stigma against khwaja sira. Khwaja sira in Swat were often estranged from their families and have repeatedly been the target of brutal homicides (Ullah et al., 2019). Some family members have engaged in physical, verbal, and emotional violence in attempts to “correct” their family member’s gender-nonconforming behaviors (Ullah et al., 2019). As stipulated by MST, the concealment of one’s identity may serve to protect individuals from expected instances of harm or violence by others (Meyer, 2003). In order to protect themselves from harmful or isolating experiences, khwaja sira may present themselves as cisgender boys/men to their families. Failure to “fully” present as men may entail their exclusion from family events, weddings, and funerals (Gupta and Murarka, 2009).

For some respondents, concealing their identities as khwaja sira provided the freedom to return to their village or participate in cultural or religious events. However, many participants reported that their family members distanced themselves in order to protect themselves from public shame or dishonor (Alizai, Ahmad and Tariq, 2017). Khwaja sira have also endured recurring sexual violence, often starting in childhood and persisting into adulthood (Azhar, Ahmad and Tariq, 2024). In Mingora, KP, khwaja sira have experienced significant physical and sexual violence, including kidnapping, robberies, beatings, and multiple gang rapes (Azhar et al., 2023). Prior research has demonstrated that the average age for the first experience of rape among khwaja sira was 12 years, marking their introduction into sexual activity (de Lind Van Wijngaarden et al., 2013).

Our findings therefore suggest that education is a primary setting where khwaja sira experience harm. In educational settings, previous research has shown that experiencing stigma, discrimination, and sexual harassment may be a primary reason that khwaja sira do not pursue higher education (Arvind et al, 2022). Many participants not only had to tolerate jeering, bullying and sexual harassment from fellow classmates and teachers, but they were also blamed by family members for instigating these acts through their “inappropriate” behavior. As stipulated by MST, we found that participants took actions to protect themselves from anticipated enacted stigma, for example, by changing their travel routes on the way to school to avoid bullies. Many khwaja sira ultimately dropped out of school to join a *dera*, a community that would be protective and inclusive of their gender identity (Azhar et al., 2022). Khwaja sira face insufficient teacher training, inadequate school facilities, and the absence of supportive school policies (Noreen and Rashid, 2024; Mehmud and Idris, 2019). These experiences contribute to their marginalization in society, leading to their denial of basic human rights (Sher et al, 2022).

In healthcare settings, third gender people are more likely to be denied access to care and to experience verbal harassment and physical violence when interacting with doctors, hospital/emergency room staff, and first responders (James et al., 2016). Individuals identifying as third gender experience pervasive discrimination in social, health, educational, and labor settings (Bhattacharya et al., 2020; Jain 2018; Ung Loh, 2018), ultimately affecting their ability to access medical care. Experiencing stigma can lead to harassment, discrimination, delay in seeking medication, and service refusal when seeking medical care (Cruz, 2014). Interactions within healthcare systems can perpetuate stigma and serve as drivers of continued vulnerability (Dolan et al., 2020). As reported in our own findings, khwaja sira are often not given priority for accessing hospital beds (Khan et al., 2020). In our study, we found that experiences of stigma and discrimination not only caused severe psychological distress but also increased the risk of poor health outcomes and untimely death. MST recognizes how sexual and gender minorities are at increased risk for mental health disorders due to the psychosocial stress resulting from stigmatization and discrimination (Meyer, 1995; Meyer, 2003).

In work settings, gender-nonbinary people often experience discrimination, harassment, and/or mistreatment (Grant et al., 2011). In Swat, khwaja sira had limited alternative employment opportunities outside dancing and sex work. Similarly, in a study of 1,366 third gender people in 3 states of India (Karnataka, Maharashtra, and Tamil Nadu), 70% of the sample reported engaging in sex work (Srivastava et al., 2023). After enduring sexual violence at school and facing stigmatization within their families and communities, it is common for khwaja sira in Pakistan to seek refuge by joining a collective home for khwaja sira, known as a*dera,* for protection (Jami and Kamal, 2015; Azhar et al., 2022). Chelas seek refuge from gender-based discrimination by affiliating with a guru in a dera (de Lind et al., 2013). Because of their perceived unemployability, many participants reported that they were unable to obtain employment in professional fields, even when they had formal training or higher education. According to MST, perceived stigma refers to the expectations for maltreatment and harassment in employment settings.

### Limitations

Our study findings are limited by our relatively small sample size and the region in which we conducted our research. Given that we conducted a qualitative study in a tribal area of Swat, Khyber Pakhtunkhwa, a region that has historically been associated with religious extremism and strict gender roles (Wang 2010), we caution against interpreting our findings to be representative of all khwaja sira in Pakistan or even all khwaja sira in the province of Khyber Pakhtunkhwa. Additionally, in order to fully protect the safety and confidentiality of our research participants, we did not collect any names, signatures, or other personally identifiable information, including age, educational background, and place of residence, potentially limiting our ability to analyze findings through these lenses of social identity. Furthermore, social desirability bias may have influenced the responses of participants during individual interviews. Due to the hierarchical nature of interactions between gurus and chelas, participants may have withheld some opinions for fear of repercussions from their gurus. Finally, in these findings, we did not report how khwaja sira communities have found ways to cope and navigate with experiences of stigma and discrimination. These findings go beyond the scope of this article and will be presented in another forthcoming manuscript focusing on resilience and coping strategies of khwaja sira.

## Social policy and practice implications

Recent social policy changes have directly impacted how sexual and gender minority groups are addressed in the Pakistani context (Khan, 2019). In 2011, the Pakistani Supreme Court passed the National Data Base Registration Act, permitting khwaja sira and other gender-nonbinary people from legally registering as a third gender on their National Identity Cards, voting registration cards, and passports (Azhar et al., 2023). In 2016, KP became the first South Asian province to implement a policy specifically aimed at protecting third gender rights (Times of Islamabad, 2016).

The Transgender Persons (Protection of Rights) Act of 2018 (“the Act”) defined “transgender” as including khwaja sira, intersex persons (*khusra, khunsa,* and *nirban*), persons assigned male at birth who have undergone sex-reassignment surgeries, or those whose gender identity or gender expression differed from the sex assigned at birth (Government of Pakistan, 2018). The Act explicitly listed inheritance, education, and the right to vote as rights, prohibited discrimination against gender-nonbinary people, and ensured access to public spaces and private property (Center for Law and Policy, 2024). According to Article 203-D of the Constitution of Pakistan, the Federal Shariat Court of Pakistan and the Islamic Ideological Council of Pakistan are tasked with determining whether a law contradicts Islamic teachings. Both institutions approved the Transgender Persons Protection of Rights Act in 2018.

In 2022 the Khunsa Persons (Protection of Rights) Bill was proposed (Senate of Pakistan, 2022) with the aim of attaining “consistency with the injunctions of Islam” (Amnesty International, 2023). The Khunsa Persons Act defines gender as an expression aligned with an individual’s assigned sex at birth (Senate of Pakistan, 2022), neglecting the distinction between gender and sex. Proposed by Senator Mushtaq Ahmad, the Khunsa Act replaced the word “transgender” with the word “khunsa.” The Act narrowly defined khunsa as someone “who has a mixture of male and female genital features or congenital ambiguities” (Amnesty International, 2023). The court referred to khunsa as a “biological infirmity,” recognizing only certain intersex characteristics that can be divided into the gender binary categories of male or female (Amnesty International, 2023). The Khunsa Act, particularly sections 14 and 20, criminalizes gender-affirming care for transgender and intersex individuals and denies protections for gender dysphoria.

On May 19, 2023, after having previously approved of the legislation, the Federal Shariat Court of Pakistan and the Islamic Ideological Council of Pakistan declared the Transgender Persons Act 2018 inconsistent with Islamic principles (Riaz and Awan, 2023). Specifically, they struck down the self-identification of gender identity, gender reassignment surgery, and the provision of inheritance rights as violating the tenets of the Qur’an. The verdict triggered a yearlong far-right propaganda campaign, leading to discussions about prohibiting the act. The previous definition of third gender under the Transgender Persons Act 2018 included khunsas, eunuchs, transgender men, transgender women, khwaja sira, or any person whose gender identity or gender expression differed from the social norms and cultural expectations based on the sex they were assigned at birth. Under the proposed Khunsa Persons Act, khwaja sira may be denied Islamic funeral and burial rights, further isolating and dehumanizing this community (Shah et al. 2018; Riaz and Awan, 2023). Critics argue that the court’s decision relies on presumptions rather than evidence, and denies essential human rights based on prejudice toward the transgender community (Amnesty International, 2023).

Despite widespread prejudice, the perspectives of khwaja sira activists have played a crucial role in shaping policy agendas and advancing progress on important sociopolitical issues, including legal rights, socioeconomic inclusion, and healthcare provision (Arslan, 2023). Urgent steps are needed to protect transgender rights, restore important provisions, and promote inclusivity in society (Riaz and Awan, 2023). Given the recent retraction of legal rights for third gender people in Pakistan, our study findings have relevance for educators, healthcare providers, and community-based practitioners who engage with khwaja sira communities.

Some effective social interventions include officially recognizing a third gender as a legal category, establishing employment quotas in public and private sectors for third gender people, and providing training and education to empower third gender individuals to be more socially included (Nisar et al., 2018). However, significant challenges remain in the Swat context, highlighting the need for targeted empowerment efforts and increased societal awareness to achieve meaningful change (Dayani et al., 2019). Educators need to be trained to be more vigilant in addressing bullying and sexual harassment within schools. Healthcare providers need to be better trained on providing nonjudgmental, gender-affirming care to their patients. Employers must work to ensure that professional work environments are inviting and nondiscriminatory for third gender people.

## Conclusion

Our research sheds light on the lived experiences of stigma and discrimination faced by khwaja sira in the Swat Valley of Khyber Pakhtunkhwa, Pakistan. Applying MST and utilizing thematic content analysis, we identified three dimensions of gender-nonconformity stigma: (1) internalized stigma, namely feelings of shame and embarrassment; (2) perceived stigma, namely opinions others had of khwaja sira regarding lack of employability or engagement in sex work; and (3) enacted stigma, namely exclusion within families, religious spaces, schools, and healthcare settings. These findings highlight the urgent need for social intervention policies to address the systemic marginalization experienced by khwaja sira communities. Results may inform future social intervention and community practice engagements aimed at promoting social inclusion of khwaja sira within Pakhtun society.

## Data Availability

The data that support the findings of this study are available from the corresponding author upon reasonable request.
