# Supplementary material for: “I would prefer to be dead than to live this way”: Lived experiences of stigma and discrimination against khwaja sira in Swat, Pakistan
Source: Glob Ment Health (Camb). 2024 Apr 30;11:e60. doi: 10.1017/gmh.2024.53 (PMC11106546; doi:10.1017/gmh.2024.53)
Supplement: Peer review history

# Editor report: Recommendation: “I would prefer to be dead than to live this way”: Lived experiences of stigma and discrimination against khwaja sira in Swat, Pakistan — R0/PR2

**DOI:** 10.1017/gmh.2024.53.pr2

**Affiliations:** The George Institute for Global Health India, India

Please respond to the comments made by reviewers. The discussion section is especially weak and needs to be rewritten using learnings from the current study and cross reference prior research as applicable.

# Editor report: Decision: “I would prefer to be dead than to live this way”: Lived experiences of stigma and discrimination against khwaja sira in Swat, Pakistan — R0/PR3

**DOI:** 10.1017/gmh.2024.53.pr3

**Affiliations:** London School of Hygiene & Tropical Medicine, United Kingdom of Great Britain and Northern Ireland

No accompanying comment.

# Editor report: Recommendation: “I would prefer to be dead than to live this way”: Lived experiences of stigma and discrimination against khwaja sira in Swat, Pakistan — R1/PR5

**DOI:** 10.1017/gmh.2024.53.pr5

**Affiliations:** The George Institute for Global Health India, India

No accompanying comment.

# Editor report: Decision: “I would prefer to be dead than to live this way”: Lived experiences of stigma and discrimination against khwaja sira in Swat, Pakistan — R1/PR6

**DOI:** 10.1017/gmh.2024.53.pr6

**Affiliations:** London School of Hygiene & Tropical Medicine, United Kingdom of Great Britain and Northern Ireland

No accompanying comment.
